# Automated Measurements of Ankle-Brachial Index: A Narrative Review

**DOI:** 10.3390/jcm10215161

**Published:** 2021-11-03

**Authors:** Aleksandra Danieluk, Sławomir Chlabicz

**Affiliations:** Department of Family Medicine, Medical University of Białystok, 15-054 Białystok, Poland; slawomir.chlabicz@umb.edu.pl

**Keywords:** peripheral artery disease, ankle-brachial index, automated ABI measurement, plethysmographic ABI, oscillometric ABI

## Abstract

Peripheral artery disease (PAD) is an atherosclerotic disease that causes obstruction in lower limb arteries. It increases cardiovascular risk even in asymptomatic patients. Accurate diagnostic tools for identification of affected individuals are needed. Recently, there have been attempts to establish a reliable method of automated ankle-brachial index (ABI) identification. A search of PubMed database to identify studies assessing automatic ABI measurements in agreement with standard PAD diagnosis methods was conducted in December 2020. A total of 57 studies were analyzed in the review. The majority of analyzed studies found ABI measured by automatic oscillometric devices to be potentially feasible for use. Some note that, even though the Doppler and oscillometric methods are not fully interchangeable, the oscillometric devices could be used in screening. Significantly fewer publications are available on automatic plethysmographic devices. For photoplethysmography, most studies reported either good or moderate agreement with reference standards. For air plethysmography, poorer agreement with Doppler ABI is suggested. It is noted that pulse volume recording (PVR) function may improve the diagnostic accuracy of the devices.

## 1. Introduction

Peripheral artery disease (PAD) is an atherosclerotic disease that causes obstruction in lower limb arteries. The prevalence of the disease among patients 40 years and older ranges from 3.1% to 5.5% [1]. The disease is more common among older patients, with rates among males at the age of 80 rising to the 20% range [2]. Prevalence of the disease is also significantly higher in diabetic patients [1]. Symptoms of PAD range from intermittent claudication to critical limb ischemia, which can lead to amputation. However, even up to 50% of the affected patients can be asymptomatic [3]. Unfortunately, the asymptomatic PAD patients also have increased cardiovascular risk [4]. Accurate diagnostic tools and efficient identification of affected individuals are therefore needed as early as at the primary care level. PAD treatment consists largely of adequate cardiovascular risk management, which can and should be introduced in the general practice. However, it has been demonstrated that PAD patients are often undertreated [5].

**Figure 1:**
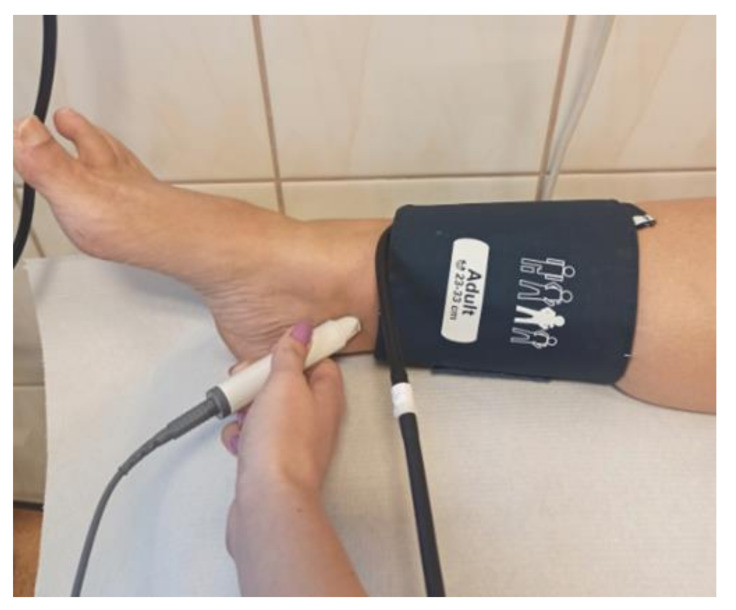
Doppler ABI measurement.

For initial PAD diagnosis, ankle-brachial index (ABI) is often used, as it is non-invasive and less expensive and more accessible than imaging studies. It can be performed at the primary care facilities to identify individuals needing further investigation. ABI is calculated by dividing systolic blood pressure at the ankle by the systolic blood pressure at the arm. The blood pressure at the ankle is traditionally taken with use of a pneumatic cuff and a continuous-wave Doppler probe (Figure 1). The cuff is placed above the subject’s ankle and inflated until the blood flow in the ankle arteries ceases, and is then deflated until a reappearance of the flow can be noted. The cessation and reappearance of the flow are manifested in the sound and visual signal produced by the Doppler probe. The reappearance of the signal is noted as systolic blood pressure at the limb. The test is non-invasive; therefore, it may also be used in the asymptomatic population, especially in high-risk groups. However, according to several studies, the test is often under-utilized or performed incorrectly in general practice [5,6,7]. The barriers for use of the ABI test as perceived by primary care personnel are time and staff constraints, availability of the equipment, and the need for training [7,8,9]. The training seems to be an important factor, given that only a short course on Doppler ABI measurement does not ensure precise ABI results [6]. The patients who could benefit from the test are therefore often referred to secondary care [5].

In the past years, there have been attempts to establish a reliable method of automated ABI identification that could prove especially useful in primary care, as it eliminates important limitations, such as time constraints and the need for personnel training. Methods such as oscillometric devices or devices using either air- or photoplethysmography (PPG) have been developed.

In the oscillometric method, a cuff is placed on the subject’s limb and then inflated until there is no blood flow in the artery recorded. Then, the cuff is slowly deflated. As the blood flow returns to the limb, blood flow starts to be recorded as oscillations in the cuff’s pressure. The maximum flow oscillations are recorded during the cuff deflation and interpreted as the average of the systolic and diastolic blood pressure, which is then used to estimate the systolic blood pressure [10].

In the photoplethysmographic method, a photosensor is used to detect the blood flow in the lower limb arteries. The sensor emits infrared light and records its reflection from the blood flow. During the measurement, a pneumatic cuff is placed above the subject’s ankle, inflated until the blood flow is fully obstructed, and then deflated. The cuff pressure at the moment when the sensor detects the reappearance of the blood flow to the artery is recorded as systolic pressure [11].

**Figure 2:**
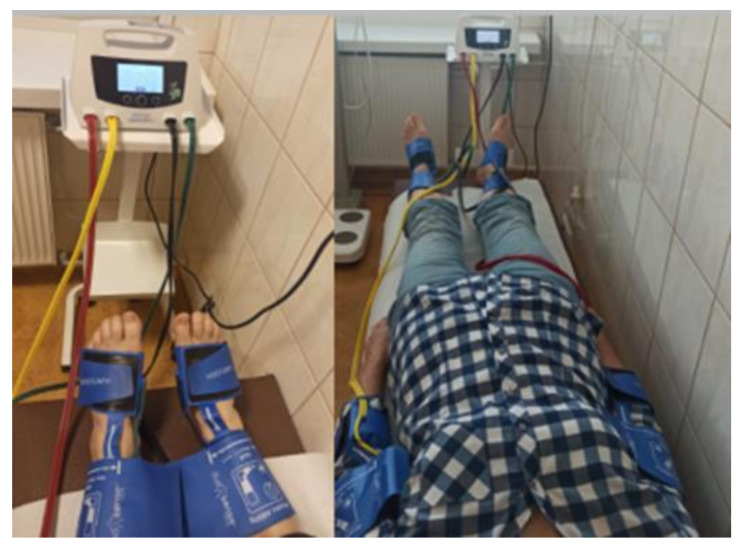
Volume plethysmography ABI measurement with use of the Dopplex ABIlity system, Huntleigh Healthcare.

In the air plethysmography method, also known as volume plethysmography, a device with dual-chamber cuffs is used (Figure 2). The upper chamber occludes the blood flow in the limb artery by increasing pressure in the cuff until it exceeds the pressure in the artery, after which it gradually decreases the cuff pressure, slowly allowing for the reperfusion. The lower chamber detects changes in limb volume with the blood flow disturbances and detects the volume increase when the blood flow through the artery is restored. The pressure in the upper chamber at the time of the blood flow restoration is recorded as systolic pressure [12].

Currently, the NICE and ESC guidelines recommend using Doppler devices over automated ABI measurement for PAD diagnosis [13,14]. AHA recommendations are in line with the aforementioned guidelines, pointing to pressure overestimation and inability to detect low pressures by oscillometric devices [15]. The same can be said of the more recent guidelines by the European Society of Vascular Medicine, which point to the fact that while alternative methods correlate well in healthy subjects, they tend to have poorer correlation in the lower range of ABI results [16]. The most recent guideline on PAD is the Asia-Pacific Consensus Statement published in August 2020, which supports ABI as a diagnostic method for PAD; however, it does not take a stance on the automatic ABI measurements, nor does it report any new advances on the topic [17]. However, given the potential for broad use of the automatic devices, especially in primary care, they are still widely studied. Some of the newly produced devices combine ABI measurement with other diagnostic modalities, such as pulse volume waveform (PVW) analysis, to improve their diagnostic ability.

To our knowledge, there have been previous reviews that aimed to assess the accuracy of oscillometric ABI devices, as well as reviews on ABI measurements in general that included studies with both oscillometric and plethysmographic ABI as the measurement method. However, there have been no reviews with plethysmographic ABI device assessment as the main aim. The last review with automatic measurement assessment as the main aim was published in 2017. Since then, new reviews have been published that included studies on automatic ABI measurements; however, their assessment was not the main goal in those works. This narrative review aims to gather available information on both of the methods, their accuracy, and agreement with the traditional diagnostic methods.

## 2. Materials and Methods

A PubMed search was conducted in December 2020, with search terms aimed to identify publications assessing the accuracy and validity of automatic ABI devices for resting ABI assessment. Terms for automatic ABI measurement, oscillometric ABI measurement, and plethysmographic ABI measurement were used, combined with terms for accuracy, validity, and Doppler ABI measurement. The full list of search terms is available in Table 1 below. The references of the papers included in the review were also screened for relevant publications. Available systematic reviews on the topic of automatic measurements were analyzed, and their results were presented in the paper.

There was no study exclusion based on publication year. Studies that analyzed automatic resting ABI measurements in agreement with any of the standard PAD diagnosis methods were included. Studies that did not perform a direct comparison to a standard method of diagnosis were excluded. Studies in languages other than English were excluded; however, where available, abstracts in English were taken into account.

## 3. Results

### 3.1. Literature Search Results

**Figure 3:**
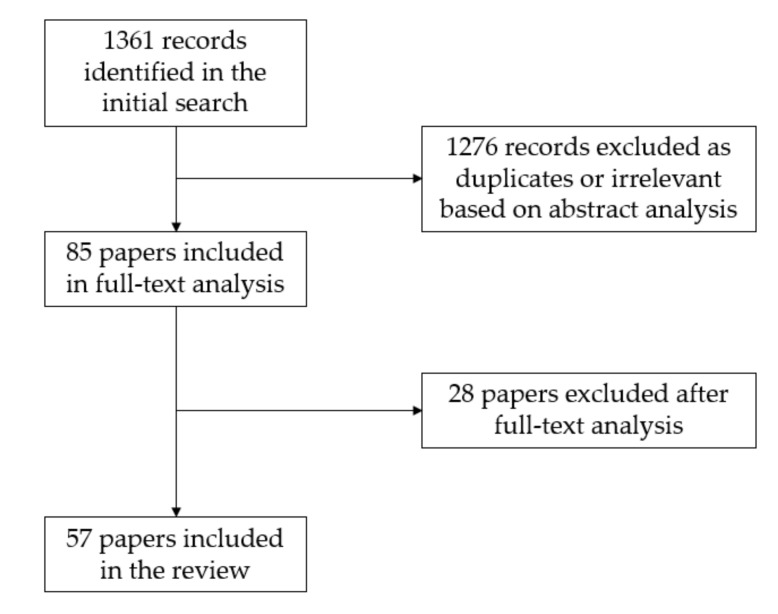
Search and study selection process.

A total of 1361 records were identified in the search, including 79 records published in 2020. Abstract analysis and duplicate exclusion showed 66 articles on oscillometric measurements and 19 articles on plethysmographic measurements that qualified for a full-text review. The reasons for record exclusion based on abstract analysis were lack of automatic ABI measurement analysis (i.e., articles that concentrated only on Doppler ABI measurements) or lack of comparison to another established diagnostic modality. After the full-text review, 22 of the studies on oscillometric devices and 5 studies on plethysmographic devices were excluded based on not meeting the eligibility criteria (Figure 3). The studies were not excluded on the basis of study type (e.g., randomized versus cross-sectional study). Language of the full text paper other than English was considered a reason for exclusion. In some instances, lack of comparison to another established diagnostic modality was not clearly established in the paper’s abstract; in those cases, the papers were excluded after full-text analysis.

### 3.2. Automatic Oscillometric Devices

Forty-three studies comparing automatic the oscillometric method to traditional PAD diagnosis underwent a full-text analysis. The vast majority of the works were cross-sectional studies, along with some case–control studies and prospective observational studies. The assessment of the validity varied among the sources. The lowest recorded sensitivity was at 20% [18], while the highest was at 97% [19]. The lowest recorded specificity was at 68.1% [20], and the highest was at 100% [21,22]. Ten of the studies did not provide data on sensitivity and specificity.

Among the publications analyzing oscillometric ABI measurements, only some of the studies used devices created specifically for ABI identification, while others used blood pressure measurement devices instead. The majority of the publications compared the results to ABI obtained by Doppler method. Other reference standards were computed tomography angiography (CTA) and ultrasound; one study performed catheter angiography on the symptomatic patients. The populations enrolled in the studies varied from the general population to vascular clinic patients or participants with cardiovascular risk factors.

The majority of analyzed studies found ABI measured by automatic oscillometric devices to be potentially feasible for use in the general population. Some noted that even though the Doppler and oscillometric methods were not fully interchangeable, the oscillometric devices could be used in outpatient screening [23,24]. In the studies that did not endorse the oscillometric ABI use, the results showed poor agreement with the Doppler technique [25,26], substantial variation of the results [26], and insufficient accuracy, especially in subjects with low ankle pressures [27].

There are important limitations to oscillometric measurements. Some of the studies show that oscillometric ABI is potentially less reliable in diabetic patients with increased vascular stiffness and in patients with very low ABI values. Ichihashi et al. found oscillometric ABI values higher than Doppler ABI at low ankle pressure, although the authors did not suggest lower diagnostic accuracy in that setting. However, the paper did indicate potentially lower diagnostic ability in diabetic patients [28]. Diehm et al. found limited correlation with Doppler ABI in diabetic patients and in critical limb ischemia [29]. In the study by Sinski et al., a trend towards larger differences between oscillometric ABI and Doppler ABI in patients with lower ABI values was found. The authors concluded that ABI is not sufficiently reliable in patients with high cardiovascular risk [30].

Some of the studies observed that oscillometry has a tendency to provide higher ABI values than Doppler [21,29,31]. However, Diehm et al. found that the correlation between measurements remained the same in the entire range of values [29]. Due to those differences, a higher cutoff point for oscillometric ABI was suggested [20,21,28,32]. For optimal sensitivity and specificity, Kollias et al. suggest a cutoff point of 0.97 [32], and in diabetic patients, Clairotte et al. suggested a cutoff value between 1.0 and 1.1 [20]. It was also suggested that when error notifications and failures to measure are considered equal to an abnormal result, the accuracy of oscillometric ABI is improved [33].

Among the potential Doppler ABI limitations that could be avoided with use of automatic measurements, time restraint seems evident. Studies that compared time needed to perform both methods found that the average time needed for automatic oscillometric measurements was significantly lower than in Doppler measurements [34,35]. In the study by Špan et al., the measurements performed by Doppler were seven times longer than the automatic ones [35]. Another existing limitation of Doppler ABI is the necessity for thorough personnel training. Vega et al. found that if the measurements were performed by inexperienced personnel, oscillometry provided more accurate values than the traditional method [19].

### 3.3. Automated Plethysmographic Devices

Fourteen studies analyzing the validity of plethysmographic ABI devices underwent full-text analysis. There were no systematic reviews or meta-analyses identified that concentrated solely on the topic of automated plethysmographic ABI measurement. The identified studies on plethysmographic devices were in majority cross-sectional or case–control studies in design. Among the studies comparing plethysmographic ABI performance with reference standards, the lowest recorded sensitivity was at 20% [36], and the highest was at 100% [37,38], although it is important to note that one of these studies used a mathematical algorithm to identify PAD individuals [37], and the other reported such a high level of sensitivity only when ABI was used in conjunction with PVW analysis and the ABI result by itself was less sensitive [38]. The lowest recorded specificity was at 76% [38]; this was a result obtained by assessing the validity of ABI in conjunction with PVW analysis. The highest recorded specificity was at 100% [37], found in a study obtaining PAD diagnosis with use of an algorithm. Six of the studies presented no data on sensitivity and specificity.

The reference tests used in the studies were either Doppler ABI or duplex ultrasonography. The populations examined in the studies were healthy subjects, patients with confirmed PAD, or subjects with known cardiovascular risk factors.

For PPG assessment, most studies reported either good or moderate agreement with the reference standard (Table 2). Teren et al. assessed PPG devices as feasible for epidemiological studies [39]. Sadiq et al. found good agreement between PPG and Doppler and endorsed the PPG method for routine use [40].

Similarly to oscillometric measurements, PPG tends to provide higher values than Doppler ABI. Teren et al. suggest that the cutoff for the abnormal ABI level should be adjusted [39]. Beutner et al. found the agreement between PPG and Doppler ABI to be better with 1.1 cutoff for PPG measurements [42].

Some of the studies analyzed time required to perform a PPG ABI assessment. While Teren et al. found no time advantage over the Doppler technique [39], Sadiq et al. found the PPG method to be quicker than the traditional one [40]. This may be due to the difference in equipment used in the studies.

It is important to note that not all devices used in the above studies for PPG ABI determination were fully automatic ABI devices; many of the papers analyzed the technique as a potential method of ABI determination.

For air plethysmography, all of the identified studies analyzed the same model of the device (Table 3). Millen et al. found that the device showed accuracy and repeatability at suboptimal levels [45], while van der Slegt et al. pointed to higher ABI values obtained with plethysmography and described the device as not applicable clinically [46]. Babaei et al. described air plethysmography specificity as excellent, along with the specificity of Doppler ABI and pulse volume waveform assessment; however, the sensitivity of plethysmography was the lowest of the three methods analyzed [36].

The studies that included PVR assessment apart from ABI measurement, found that component to be more diagnostically accurate than ABI measurement alone [36,38,45]. Lewis et al. concluded that combining both methods is a highly accurate PAD exclusion modality [38].

Some of the studies suggested that while air plethysmography devices cannot be considered a standalone PAD diagnostic method, they might prove useful for identifying individuals needing further assessment. They suggested using a higher ABI value cutoff for that purpose [12,36].

## 4. Discussion

### 4.1. Accuracy of Automated Devices

The current PAD guidelines do not recommend the general use of automatic devices, potentially because summarizing all available information does not give a clear answer on their applicability in clinical settings.

While there is a good number of publications on oscillometric ABI devices, the results obtained by the researchers varied from finding good agreement to weak agreement with reference standards. It is important to analyze the reason for such differences in the results and whether potential limitations in oscillometric ABI devices should exclude them from general use.

Interestingly, in the studies that do not endorse oscillometric ABI measurements in clinical practice, either a standard blood pressure measuring device was used instead of an ABI targeted one, or the population consisted of vascular or cardiovascular clinic patients. Only one study that found poor agreement with the reference standard did not have any of the above features; however, its population consisted of coronary artery disease patients [30]. Less accurate results in the aforementioned studies may be due to the fact ABI targeted devices have the potential to provide better diagnostic results than regular blood pressure devices, possibly due to simultaneous measurements in the former. Furthermore, the vascular clinic patient population typically presents with lower ABI levels, and oscillometric ABI has a tendency to be less reliable in low ABI ranges [21,31,48]. Diagnostic accuracy of the device can vary in specific populations. A systematic review by Herráiz-Adillo et al. showed higher sensitivity of oscillometric ABI measurements in vascular services and higher specificity in primary care [49] Some studies conclude that while the oscillometry ABI agreement with the reference standard is weak, it could still be used in screening [23,24]. Further studies are needed to evaluate whether device refinements, cutoff adjustments, and limitations of the population qualified for testing will be enough to use oscillometric ABI devices as reliable testing alternatives.

The most recent systematic review and meta-analysis that concentrated solely on assessing automatic ABI accuracy against reference tests was published in 2017 and yielded positive results, deeming oscillometric ABI as accurate and feasible enough to be useful for PAD diagnosis [49]. The latter was connected to the shorter time needed to perform measurements and a shorter learning curve. The meta-analysis found that global sensitivity and specificity of oscillometric ABI measurements in analyzed studies were 65% and 96%, respectively. It also pointed to lower accuracy of measurements in diabetic patients [49]. Since then, a systematic review and meta-analysis on ABI and TBI accuracy has been published, including studies that performed automatic ABI measurements as well. The review points out that in a subgroup analysis of automated and Doppler ABI, similar diagnostic accuracy was found. In the meta-analysis, automatic ABI sensitivity and specificity against reference standard tests were 62% and 92%, respectively [50]. Another systematic review on general ABI reliability was published in 2019, and it reported three studies that used automatic measurements: one with a plethysmographic device, one with an oscillometric device, and one with ABI measured with Doppler probe and TBI measured by means of plethysmography. The review noted marginally better reliability in automatic measurements compared to the manual ones [51]. Furthermore, the Cochrane Database systematic review on both oscillometric and Doppler measurements of ABI suggests that automatic oscillometric measurement may even be more accurate than Doppler when used by untrained individuals. However, based on the selection criteria, the review only included one study [52]. Another systematic review and meta-analysis reports that while values provided by oscillometric measurements have a tendency to be higher, they still appear to be feasible and accurate [53]. Since the last systematic review with automatic ABI measurement assessment as the main aim, several new works have emerged, with a great majority of them concluding that automatic oscillometric ABI is a reliable tool, especially in primary care settings [48,54,55,56,57,58]. This could show that with time and advances in automatic ABI device development, this diagnostic modality has become more refined, and thus, a larger proportion of the studies have begun to find it feasible in clinical care. Among the papers assessing oscillometric ABI feasibility published from 2017 forward, only one study, by Homza et al., found automatic measurements to be suboptimal and useless for screening due to low sensitivity and poor negative predictive value [59]. The study was performed exclusively on diabetic patients, and it could be concluded that the specific population was the reason for the negative result, especially since previous studies have shown that a lower accuracy is to be expected in the diabetic population. However, two other recent studies on diabetic patients have shown oscillometric ABI to be reliable even in that population [34,48], so the question arises whether the results might be device-dependent or other variables have come into play. Another interesting study was published in 2019, analyzing the capacity of oscillometric ABI to predict all-cause mortality rather than comparing it to Doppler ABI solely as a PAD diagnosis tool. The study showed that the abnormal result of oscillometric ABI measurement was predictive for all-cause mortality with higher capacity than abnormal Doppler ABI, independently from cardiovascular risk factors [60].

Assessment of automatic plethysmographic ABI devices is more difficult, with a sparse amount of material available on the subject. PPG seems to be promising, with many of the studies reporting good or moderate agreement with reference standards. It seems that PPG might pose the same sort of difficulties as oscillometric measurements. Studies show higher ABI values obtained in PPG than in Doppler, so an adjustment of the ABI cutoff might be needed [39,42]. There is almost an equally limited amount of material on air plethysmography. Some of the studies show air plethysmography as less reliable than other methods, with two publications endorsing it only for the initial identification of at-risk individuals [12,36]. However, especially in the primary care setting, such limited use might still prove beneficial. Additionally, some of the air plethysmography devices also offer PVR assessment, which is a factor improving diagnostic accuracy [38].

### 4.2. Resting ABI limitations

When standardized methodology is applied, resting ABI is considered the first-line diagnostic test for PAD, and its diagnostic performance in detecting >50% stenosis is considered reasonably good. However, it is important to note that while resting ABI specificity is consistently high, standing at 83 to 96% in different studies, sensitivity varies and is considerably lower in most studies, standing at 61 to 73% [61].

It has been observed in previous studies that resting ABI sensitivity may be limited by several factors. ABI results can be falsely elevated in the presence of artery calcification, which makes the vessels less compressible, for example, in patients with diabetes or chronic kidney disease [62].

Sensitivity of the test also depends on the ABI threshold used to diagnose PAD. It is generally agreed that the basic threshold for PAD diagnosis is an ABI result lower than 0.9; however, the values between 0.9 and 1.0 are considered borderline and should be interpreted along with the information on the clinical probability of the disease, or ideally, confirmed with another test as they do not rule out the possibility of disease [15].

Post-exercise ABI measurement, performed after a treadmill test or repeated plantar flexion, improves ABI sensitivity, and can be particularly useful in cases of borderline resting ABI and in patients with normal ABI presenting with typical PAD symptoms [61]. A study by Mahe et al. observed that in the borderline resting ABI patient group, one third of the patients had an abnormal postexercise ABI result [63]. In the case of post-exercise measurements of ABI in healthy patients, a mild decrease in ABI level is observed immediately after exercise, followed by a rapid increase to normal values in the following 1–2 min. In the presence of PAD, the post-exercise ABI level decrease is more prominent and lasts longer than in the healthy patients [15]. The current AHA criteria for postexercise ABI measurements diagnostic for PAD are a postexercise decrease in blood pressure higher than 30 mm Hg or ABI decrease higher than >20% [61]. Recent studies show that these criteria might not be optimal in terms of sensitivity, given that an exercise ABI of less than 0.9 has an 88% sensitivity to detect >50% and >75% stenoses, higher than either of the criteria proposed by AHA. However, there is a significant trade-off in specificity to be considered, with exercise ABI < 0.9 specificity to detect stenosis found at 26% and 31% for 75% and 50% stenosis, respectively [64]. Nevertheless, exercise ABI allows for the correct diagnosis of a higher number of PAD cases than the resting ABI [64].

Furthermore, in patients with potentially increased arterial stiffness by calcification, the toe-brachial index (TBI) may be a more suitable diagnostic test than ABI. Studies show that its diagnostic accuracy does not differ in patients with diabetes versus patients without diabetes [62].

## 5. Conclusions

Even though there are important limitations to consider, many of the studies on oscillometric measurements find them feasible and correlating with previous diagnostic modalities. The PPG method seems promising; however, few studies have analyzed fully automatic PPG ABI devices. Future studies may give clinicians more insight into PPG feasibility. Air plethysmography was the least reliable of the analyzed methods; however, the devices with PVR assessment function could be useful in initial screening.

In the analysis of feasibility of a device, it is important to take the setting in which it will be mostly used into account. Automatic ABI devices will mostly be needed in settings such as primary care, where the personnel are normally not well acquainted with performing specialized diagnostic methods. In such settings, adequate initial assessment of the broad population might be more important than identification of the stages of the disease or decision on borderline cases. A device that is only feasible for screening, which would be suboptimal in a specialized setting, still can be very useful in primary care. Further studies might show the exact populations and clinical situations in which automatic ABI measurements are applicable.

## Figures and Tables

**Table 1:** Full list of search terms used in the review.

No	Search Term
1	oscillometric ABI
2	oscillometric ankle-brachial index
3	plethysmographic ABI
4	plethysmographic ankle-brachial index
5	automated ABI
6	automated ankle-brachial index
7	automatic ABI
8	automatic ankle-brachial index
9	doppler ABI vs. automatic ABI
10	doppler ankle-brachial index vs. automatic ankle-brachial index
11	doppler ABI vs. oscillometric ABI
12	doppler ankle-brachial index vs. oscillometric ankle-brachial index
13	doppler ABI vs. plethysmographic ABI
14	doppler ankle-brachial index vs. plethysmographic ankle-brachial index
15	automatic ABI validation
16	automatic ankle-brachial index validation
17	oscillometric ankle-brachial index validation
18	plethysmographic ankle-brachial index validation
19	automatic ABI validity
20	automatic ankle-brachial index validity
21	oscillometric ankle-brachial index validity
22	plethysmographic ankle-brachial index validity
23	doppler ABI vs. automated ABI
24	doppler ankle-brachial index vs. automated ankle-brachial index,
25	automated ABI validity
26	automated ankle-brachial index validity
27	automated ABI validation
28	automated ankle-brachial index validation
29	doppler ABI vs. plethysmography ABI
30	doppler ankle-brachial index vs. plethysmography ankle-brachial index
31	plethysmography ankle-brachial index validation
32	plethysmography ankle-brachial index validity

**Table 2:** Studies on photoplethysmography ABI devices. Abbreviations: ABI—ankle-brachial index, PPG—photoplethysmography.

No.	Author/Year	Reference	Device	Sensitivity	Specificity	Author Conclusions
1	Arnold et al., 2020 [41]	Doppler ABI	Masimo Rad-97	n/a	n/a	High level of agreement.
2	Teren et al., 2013 [39]	Doppler ABI	Vascular Explorer and Vicorder	n/a	n/a	Moderate concordance with Doppler ABI. Tendency for higher values in PPG than Doppler ABI.
3	Beutner et al., 2012 [42]	Doppler ABI	Vascular Explorer and Vicorder	75%, 85%, 80% ^1^	96%, 89%, 98% ^1^	Excellent diagnostic value. Tendency for higher values in PPG than Doppler ABI.
4	Khandanpour et al., 2009 [11]	Doppler ABI	Viasys VasoGuard MicroLite	n/a	n/a	A promising alternative to Doppler.
5	Alnaeb et al., 2008 [43]	Doppler ABI and duplex scan	Custom PPG probe	86%	85%	Can be used to identify patients at risk.
6	Alnaeb et al., 2007 [44]	Doppler ABI and duplex scan	Custom PPG probe	83%	71%	Promising technique for diabetic patient assessment.
7	Jönsson et al., 2005 [37]	Doppler ABI	Custom PPG probe	100%	100%	Further elaboration of the technique is motivated.
8	Sadiq et al., 2001 [40]	Doppler ABI	Healthwatch	n/a	n/a	Recommended to use on routine basis.

^1^ Depending on device and deflationary/inflationary method.

**Table 3:** Studies on air plethysmography ABI devices. Abbreviations: ABI—ankle-brachial index, PVW—pulse volume waveform, PAD—peripheral artery disease.

No.	Author/Year	Reference	Device	Sensitivity	Specificity	Author Conclusions
1	Babaei et al., 2019 [36]	Doppler ABI	Dopplex Ability	20%, 40% ^1^	95.6%, 79.9% ^1^	Not sufficient as standalone test. Potentially useful for identifying individuals needing further assessment after adjusting cutoff value. The study also analyzed PVW qualitative assessment, which was found to be more effective than ABI.
2	Millen et al., 2018 [45]	Doppler and plethysmography based ABI device	Dopplex Ability	59% ^2^	86% ^2^	Not accurate. The study also analyzed PVW qualitative assessment, which was found to be more effective than ABI.
3	Lewis et al., 2016 [38]	Duplex scan	Dopplex Ability	79%	91%	When combined with PVW analysis, can be highly accurate to rule out PAD.
4	Van der Slegt et al., 2016 [46]	Doppler ABI	Dopplex Ability	n/a	n/a	Not applicable in post-operative measurements.
5	Davies et al., 2015 [12]	Doppler ABI	Dopplex Ability	70%, 98% ^3^	96%, 75% ^3^	Unclear whether it can be used as standalone method. Potentially useful for identifying individuals needing further assessment after adjusting cutoff value.
6	Lewis et al., 2010 [47]	Doppler ABI	Dopplex Ability	n/a	n/a	Potential for PAD screening in primary care.

^1^ With 1.2 cutoff for plethysmography. ^2^ Respectively 56% and 82% according to the abstract. ^3^ With 1.04 cutoff for plethysmography.

## Data Availability

Not applicable.
